# Czech and Slovak Dental Students’ Oral Health-Related Knowledge, Attitudes, and Behaviours (KAB): Multi-Country Cross-Sectional Study

**DOI:** 10.3390/ijerph19052717

**Published:** 2022-02-25

**Authors:** Abanoub Riad, Veronika Chuchmová, Ján Staněk, Barbora Hocková, Sameh Attia, Martin Krsek, Miloslav Klugar

**Affiliations:** 1 Czech National Centre for Evidence-Based Healthcare and Knowledge Translation (Cochrane Czech Republic, Czech EBHC: JBI Centre of Excellence, Masaryk University GRADE Centre), Faculty of Medicine, Institute of Biostatistics and Analyses, Masaryk University, 625 00 Brno, Czech Republic; 2 Department of Public Health, Faculty of Medicine, Masaryk University, 625 00 Brno, Czech Republic; veronika.chuchmova@med.muni.cz (V.C.); krsek@med.muni.cz (M.K.); 3 Department of Prosthetic Dentistry, Faculty of Medicine and Dentistry, Palacký University Olomouc, 775 15 Olomouc, Czech Republic; stanekjano@gmail.com; 4 Department of Maxillofacial Surgery, F. D. Roosevelt University Hospital, 975 17 Banska Bystrica, Slovakia; bhockova@nspbb.sk; 5 Department of Oral and Maxillofacial Surgery, Justus-Liebig-University, Klinikstrasse 33, 35392 Giessen, Germany; sameh.attia@dentist.med.uni-giessen.de

**Keywords:** Czech Republic, dental education, dental students, Slovakia, health-related knowledge, attitudes and practices, Hiroshima University Dental Behavioural Inventory, HU-DBI, oral health, oral hygiene

## Abstract

Dentists play a key role in the primary prevention of oral diseases and related systemic complications; therefore, their views on behavioural interventions need to be aligned with the current agendas for oral health. Likewise, dental students’ oral health-related knowledge, attitudes, and behaviours (KAB) are of practical importance, as they are the future opinion leaders for oral health in their respective communities. A cross-sectional survey-based study was designed to evaluate the oral health KAB of dental students in both the Czech Republic and Slovakia. The study utilized translated versions of the Hiroshima University Dental Behavioural Inventory (HU-DBI), and it aimed to recruit students from all Czech and Slovak dental schools. A total of 487 students were included in this study, out of which 372 (76.4%) were females, 271 (55.6%) were enrolled in preclinical years, 68 (14%) reported smoking tobacco at least once a week, and 430 (88.3%) reported problematic internet use. The mean HU-DBI score of Czech and Slovak dental students (8.18 ± 1.80) was comparable with the previously reported scores of dental students in Nordic and Western European countries. Czech students (9.34 ± 1.29) had a significantly higher score than their Slovak counterparts (7.56 ± 1.73). In both countries, preclinical students (8.04 vs. 8.35), the students who reported tobacco smoking (7.63 vs. 8.27), and those who reported problematic internet use (8.11 vs. 8.70) had significantly lower HU-DBI scores than their counterparts, respectively. In the Czech Republic, the significant increases in HU-DBI scores occurred after the first academic year when the students received preventive dentistry courses; therefore, one can put forward that early implementation of preventive elements in undergraduate dental curricula may yield better and more sustainable oral health gains for the students. Future research on Czech and Slovak dental curricula need to re-evaluate the oral hygiene and anti-smoking components and their impact on students’ views and attitudes.

## 1. Introduction

In May 2021, the World Health Organization (WHO) undertook a historic step by approving a resolution on oral health that incorporates oral health within the vision of 2030 for non-communicable diseases (NCDs) [1]. The WHO member states are urged now to address the modifiable risk factors of oral diseases that are shared with non-communicable diseases, such as free sugar intake and tobacco use [1,2]. Besides the fact that oral diseases are the most prevalent NCDs globally today, the importance of oral health to systemic health is underlined by a myriad of pathophysiologic interactions between oral and systemic diseases, e.g., diabetes mellitus, cardiovascular disease, and malignancies [3,4,5].

Oral diseases are multi-factorial in nature, even though it has been well-established that all patients’ involvement in oral health is entirely behavioural [6,7,8]. The primary prevention of oral diseases implies multiple behavioural targets such as twice-daily toothbrushing, periodic dental check-ups, sugar intake reduction, and smoking cessation, which require multi-level and multi-sectorial approaches to be achieved [3].

Dentists and dental teams’ members have a vital role in this game, as they can provide professional advice to their patients for maintaining good oral hygiene [9,10]. Multiple systematic reviews have recently shown that there is convincing evidence on the immediate effect of educational and promotional interventions in oral health, which justify the need for more active and incentivized roles of dentists and dental hygienists in behavioural counselling [11,12,13,14,15,16]. Likewise, dental students are the future opinion leaders of oral health in their communities; therefore, their oral health-related knowledge, attitudes, and behaviours (KAB) can reflect their self-care views and indicate how much they may be willing to perform behavioural interventions [17,18,19]. Given the public perceptions of physicians and dentists as exemplary models for healthy lifestyles, the promotional roles of dentists are not limited to teaching proper brushing techniques, but they can be extended to include other behavioural targets, e.g., tobacco cessation, moderate alcohol consumption, physical activity, healthy nutrition, and immunization [20,21,22,23,24,25,26,27,28].

In the Czech Republic and Slovakia, the lack of national strategies for oral health is a stumbling block to meeting the targets set by the European and international entities [29]. The Czech oral healthcare system is primarily dependent on private providers and cost-sharing models where the insurance companies are obliged to cover basic preventive and therapeutic services [30]. However, preventive services such as regular check-ups are remunerated by the current packages, and the amount of out-of-pocket expenditures has increased significantly during the last twenty years [30]. Similarly, public insurance covers regular check-ups in Slovakia, with recent initiatives aimed at complementing these preventive services by restoring school visiting programs [31]. Moreover, preventive dentistry has been included in undergraduate dental curricula for a long time, and it is one of the core competencies for trained dental professionals in both countries [32].

The Hiroshima University Dental Behavioural Inventory (HU-DBI) developed by Kawamura in 1988 is a psychometric instrument which is widely used to evaluate oral health-related KAB among dental students [33]. Thanks to its psychometric properties, limited length and filling time, and its multi-dimensionality, the HU-DBI had been translated and culturally adapted to multiple languages; therefore, an international comparison of nationally collected data is deemed feasible [34]. The instrument had been tested in various contexts, and it was found to have good capacity to predict clinical outcomes [35].

The overall aim of this study was to evaluate the oral health KAB of dental students in the Czech Republic and Slovakia. The primary objective was to estimate the levels of oral health KAB using HU-DBI among dental students in the Czech Republic and Slovakia. The secondary objectives were (i) to assess the role of gender, academic level and clinical experience on students’ oral health KAB, and (ii) to explore the association between oral health KAB and risk health behaviours, e.g., tobacco smoking, alcohol drinking, and problematic internet use.

## 2. Materials and Methods

### 2.1. Design

A cross-sectional survey-based study was carried out during the autumn semester of the academic year 2021/2022 utilizing a self-administered questionnaire (SAQ) to collect data from dental students in the Czech Republic and Slovakia. The SAQ was coded and disseminated digitally using KoBoToolbox (Harvard Humanitarian Initiative, Cambridge, MA, USA, 2021) [36]. A secured unique resource locator (URL) was used in data collection where no repetitive filling of the questionnaire was possible from the same internet protocol (IP) address. The study was reported according to the STrengthening the Reporting of OBservational studies in Epidemiology (STROBRE) guidelines for cross-sectional studies [37]

### 2.2. Participants

The target population of this study were undergraduate dental students in the Czech Republic and Slovakia who were enrolled as full-time students during the academic year 2021/2022. The international students enrolled in English programs were not included in this study, nor were Erasmus students. The master’s degree program of dentistry lasts for five years in the Czech Republic and six years in Slovakia [32,38]. The first three years in Czech and Slovak curricula are predominantly occupied by basic medical and dental sciences; therefore, the first, second, and third year are considered preclinical years. On the other hand, the curricula of the following years: fourth and fifth year in the Czech Republic; and fourth, fifth, and sixth year in Slovakia are occupied by clinical dentistry courses; therefore, they are considered clinical years [32,38].

The target participants were invited through multiple channels: (i) a mass email was sent to the members’ list of the Slovak Association of Dental Students (Slovenský Spolok Študentov Zubného Lekárstva “SSŠZL”), and (ii) promotional posts were published at Facebook groups of dental students in the Czech Republic [39]. The participants who did not complete the survey or those who did not indicate their informed consent digitally at the beginning were excluded from the final analyses.

According to the latest report of the Slovak Dentists Chamber (Slovenská Komora Zubných Lekárov “SKZL”), the total number of dental students enrolled in Slovak universities was 674 students in 2020 [40,41]. The total number of dental students in the Czech Republic was estimated to be ≈1800 students [42]. The sample size required for this study was computed using Epi-Info ^TM^ version 7.2.5 (CDC. Atlanta, GA, USA, 2021) through the “Population Survey” module, following the assumptions that the confidence level would be 95%, error margin would be 5%, number of clusters would be 2, and expected frequency would be 50% [43,44]. The required sample was 167 students in each country, which is equal to 333 students overall.

**Figure 1:**
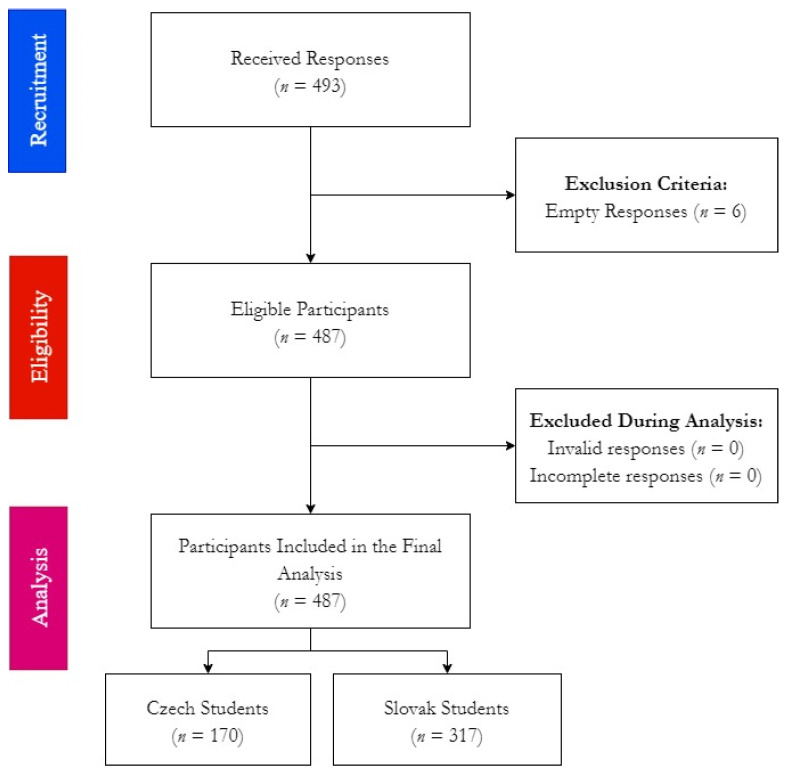
Workflow of HU-DBI survey among Czech and Slovak dental students, Autumn 2021.

A total of 493 responses were received from the target population, four Slovak responses and two Czech responses were empty, and they were excluded from the study. None of the eligible responses had missing or invalid data; therefore, the remaining 487 responses were included in the final analysis (Figure 1).

### 2.3. Instrument

The SAQ comprised three main categories: (i) demographic characteristics including gender, university, and academic level, (ii) the original HU-DBI items (*n* = 20), and (iii) general health behaviours including tobacco smoking “I consume tobacco at least once a week”, alcohol drinking “I drink alcohol at least once a week”, problematic internet use “I find myself using my smartphone/compute longer than I planned”, and regular dental check-ups “I go to the dentist/ hygienist for a regular check-up at least once a year” [45,46,47] (Appendix A).

#### 2.3.1. HU-DBI Scoring System

The original HU-DBI instrument had twenty dichotomous (Agree/Disagree) items that are used to evaluate oral health-related knowledge (items no. 2, 8, 10, 15, and 19), attitudes (items no. 9, 11, and 14), and behaviours (items no. 4, 9, 12, 16) [17]. The overall score of HU-DBI is based on the sum of twelve core items; therefore, it ranges between 0 and 12, where the higher score indicates better overall oral health KAB. For the final HU-DBI score, one point is given for each “agree” answer of items no. 4, 9, 11, 12, 16, and 19, and each “disagree” answer of items no. 2, 6, 8, 10, 14, and 15.

#### 2.3.2. Czech HU-DBI

The guidelines of Beaton et al. 2000 for translation and cross-cultural adaptation had been followed for producing a validated Czech version of HU-DBI [48]. Firstly, forward translation from English to Czech had been performed by two independent translators (FT1 and FT2) whose first language was Czech, and both of them had a dental background. Then, an experts’ panel was formed to review the two Czech versions (FT1 and FT2) and produce a common version (FT–12) which was used in the third stage, “backward translation”. Two translators (BT1 and BT2) whose first language was English had been invited to translate the FT–12 from Czech to English independently. In the fourth stage, another experts’ panel comprising the four translators and the study investigators was formed to review BT1, BT2, FT–12, and original English HU-DBI versions in order to discuss all the linguistic and grammatical discrepancies with the intention of producing a pre-final Czech version.

The pre-final Czech version had undergone two phases of psychometric testing to verify its bi-lingual reliability (preliminary testing) and test–re-test reliability (final testing). The preliminary testing phase involved a random sample of 20 young Czech individuals who had a good proficiency level of the English language who were invited to fill in the English version of HU-DBI primarily, and after 24 h, they filled in the pre-final Czech version. Cognitive debriefing (interviews) was conducted by asking 10 out of the 20 volunteers who participated in preliminary testing to share their feedback about the clarity and equivalence of the Czech translation and their suggestions to improve it. The minimum inter-rater agreement level was set to be 80%; therefore, any item rated as unclear by at least 20% of the volunteers, would have been referred back to the expert panel for further consultation and adaptation.

The final phase of psychometric testing (test–re-test reliability) was carried out by inviting a random sample of 40 Czech university students to fill in the pre-final Czech version twice with an interval of 48 h, recommended by Marx et al. 2003 [49]. The mean Cohen’s kappa coefficient (*κ*) was 0.941 ± 0.070, and it ranged between 0.754 (item no. 1) and 1.000 (items no. 5, 6, 7, 15, 16, 18, 19, and 20). According to McHugh criteria for interpreting the Cohen’s *κ* coefficient, the Czech HU-DBI version had an almost perfect level of reliability [50] (Supplementary Table S1).

#### 2.3.3. Slovak HU-DBI

The WHO guidelines for translation and cross-cultural adaptation had been used in producing the Slovak HU-DBI version [51]. The WHO guidelines were pragmatic and involved forward translation by two Slovak native translators (from English to Slovak) and backward translation by a single translator (from Slovak to English). All translators were healthcare professionals. Then, an expert panel was formed to review the produced versions and compare them to the original English HU-DBI version in order to relieve linguistic and grammatical issues. Psychometric testing involved five students who were asked about their opinion about the clarity and equivalence of the Slovak translation to the English source.

Eventually, two items were found to be non-comparable between Czech and Slovak versions; therefore, cross-country comparison of those two items (no. 1 and no. 5) should be approached with caution. The verb “worry” in item no. 1 was translated as “fear” in the Czech version, while the Slovak version used its synonym “concern”. The term “child-sized toothbrush” in item no. 5 was literally translated in the Slovak version, while the Czech version simplified it as “small-headed toothbrush” (Appendix A).

### 2.4. Ethics

The Ethics Committee of the Faculty of Medicine, Masaryk University reviewed and approved the protocol of this study on 20 November 2019 (Ref no. 48/2019). The declaration of Helsinki for research involving human subjects and the European Union (EU) general data protection regulation (GDPR) guided the design and execution of the present study [52,53]. All participating students had to indicate their consent digitally prior to their participation, and those who failed to indicate their consent were disqualified from the study. No identifying personal data was collected; therefore, retrospective identification of the participants was not possible. Participation in this study was not encouraged by any means of incentives, and it was not coerced by any means of penalties.

### 2.5. Analyses

Initially, Shapiro–Wilk test had been performed to verify whether the overall HU-DBI score (0–12) and its subdomains, i.e., knowledge (0–5), attitudes (0–3), and behaviours (0–4) were normally distributed or not with a significance level (*Sig*.) < 0.05. The HU-DBI scores of Czech and Slovak dental students were not normally distributed; therefore, the non-parametric analytical tests were used.

Descriptive statistics for the nominal variables (gender and country), ordinal variables (academic level and HU-DBI items answers), and numerical variables (HU-DBI scores) had been executed using frequencies (*n*) and percentages (*%*) for qualitative variables, and mean and standard deviations (*µ* ± *SD*) for quantitative variables. Inferential statistics had been executed to test the association between HU-DBI responses and scores and sociodemographic and behavioural correlates. Chi-squared test (*χ*^2^), Fisher’s exact test, Mann–Whitney test (*U*), and Jonckheere-Terpstra test (*JT*) were used with a confidence level (*CI*) of 95% and a significance level (*Sig*.) < 0.05.

Binary logistic regression had been performed on the dependent variable (country); and it estimated the adjusted odds ratio (AOR) of the HU-DBI core items and the sociodemographic and behavioural predictors, which were found to be significant in the univariate analysis (Chi-squared test (*χ*^2^) and Fisher’s exact test). The Nagelkerke pseudo R^2^ was used to explain the variability of group membership (country). Similarly, logistic regression analysis was used to evaluate the predictors of tobacco smoking behaviours.

## 3. Results

### 3.1. Demographic Characteristics

Out of the 487 students who were included in the downstream analyses, 372 (76.4%) were females and represented 73.5% and 77.9% of Czech and Slovak samples, respectively, without a statistically significant difference (*Sig*. = 0.277). Over half of the participants (55.6%) were enrolled in preclinical years without a statistically significant difference (*Sig*. = 0.909) between Czech (55.3%) and Slovak (55.8%) samples (Table 1).

From the Czech Republic, 170 students were included with the Faculty of Medicine and Dentistry, Palacký University Olomouc being the most contributing faculty (79.4%), followed by the Faculty of Medicine, Masaryk University (10%), and the First Faculty of Medicine, Charles University (5.9%).

From Slovakia, 317 students were included with Jessenius Faculty of Medicine in Martin, Comenius University being the most contributing faculty (32.2%), followed by the Faculty of Medicine, Pavol Jozef Šafárik University (29.7%), the Faculty of Medicine in Bratislava, Comenius University (24.3%), and the Faculty of Medicine, Slovak Medical University in Bratislava (13.9%).

### 3.2. Health Behaviours

Tobacco smoking at least once a week was reported by 68 (14%) students, and it was significantly (*Sig*. = 0.008 and <0.001) more common among Slovak (17%) and male students (24.3%) than their Czech (8.2%) and female colleagues (10.8%), respectively. Drinking alcohol at least once a week was reported by more than one-third of the participants (35.5%), with males having a significantly (*Sig*. < 0.001) higher prevalence (50.4%) than females (30.9%) in both countries.

The majority of participants (88.3%) reported problematic internet use, which was more common (*Sig*. = 0.017) among Slovak (90.9%) than Czech (83.5%) students. Regular dental check-ups annually were also reported by the vast majority of our participants (93.6%) with no statistically significant differences based on country, gender, or clinical experience (Table 2).

### 3.3. HU-DBI Responses

Among Czech students, item no. 3 of worrying about teeth colour received the highest level of agreement (94.1%), followed by item no. 1 of dental anxiety (91.2%), and item no. 5 of using child-sized toothbrushes (90%). Contrarily, item no. 2 of bleeding gingiva had the lowest level of agreement (0.6%), followed by item no. 17 of using toothbrushes with hard bristles (1.8%), item no. 7 of dissatisfaction with gingival colour (2.4%), and item no. 6 of incapacity to maintain oral health in older age (2.9%).

Among Slovak students, item no. 3 of worrying about teeth colour received the highest level of agreement (96.2%), followed by item no. 9 of careful toothbrushing (80.1%), and item no. 12 of post-brushing checking (79.2%). Contrarily, item no. 5 of using child-sized toothbrushes had the lowest level of agreement (3.8%), followed by item no. 2 of bleeding gingiva (8.8%), item no. 15 delaying dental visits (8.8%), item no. 7 of dissatisfaction with gingival colour (10.4%), and item no. 17 of using toothbrushes with hard bristles (10.4%).

The difference between Czech and Slovak students was statistically significant in fifteen items. Slovak students exhibited significantly higher agreement levels for items no. 2 of gingival bleeding (8.8% vs. 0.6%), no. 4 of noticing dental plaque (31.9% vs. 16.5%), no. 6 of incapacity to maintain oral health in older age (30.3% vs. 2.9%), no. 7 of dissatisfaction with gingival colour (10.4% vs. 2.4%), no. 8 of perceived-efficacy of oral hygiene (20.2% vs. 6.1%), no. 10 of receiving professional oral hygiene training (25.6% vs. 5.9%), no. 14 of preventing periodontal of toothbrushing solely (34.1% vs. 15.3%), no. 17 of using a toothbrush with hard bristles (10.4% vs. 1.8%), and no. 18 of aggressive toothbrushing (14.5% vs. 3.5%) than Czech students. On the other hand, Slovak students exhibited significantly lower agreement levels for items no. 11 of toothbrushing without toothpaste (37.5% vs. 84.1%), no. 16 of using plaque-disclosing agents (37.2% vs. 70%), and no. 19 of spending too much time while toothbrushing (18% vs. 52.9%) than Czech students (Table 3).

#### 3.3.1. Academic Level

In the Czech Republic, the fifth-year students (seniors) had significantly higher agreement levels for items no. 1 of dental anxiety (93.8% vs. 69.2%), no. 11 of toothbrushing without toothpaste (91.7% vs. 53.8%), and no. 16 of using plaque-disclosing agents (83.3% vs. 53.8%) than the first-year students (freshers), respectively. On the other hand, freshers had significantly higher agreement levels for items no. 6 (15.4% vs. 0%), no. 10 of receiving professional oral hygiene training (23.1% vs. 4.2%), no. 12 of post-brushing checking (92.3% vs. 60.4%), and no. 17 of using toothbrushes with hard bristles (15.4% vs. 0%) than seniors, respectively.

In Slovakia, the sixth-year students (seniors) had significantly higher agreement levels for items no. 9 of careful toothbrushing (92.6% vs. 75%), no. 11 of toothbrushing without toothpaste (66.7% vs. 19.4%), no. 13 of worrying about halitosis (55.6% vs. 23.6%), and no. 16 of using plaque-disclosing agents (63% vs. 33.3%), than the first-year students (freshers), respectively. On the other hand, freshers had significantly higher agreement levels for items no. 17 of using toothbrushes with hard bristles (18.1% vs. 0%) and no. 18 of aggressive toothbrushing (25% vs. 7.4%) than seniors, respectively (Table 3).

#### 3.3.2. Gender

On comparing HU-DBI responses across genders, item no. 3 of worrying about teeth colour was significantly more common among females (97.6%) than males (88.77%) in both countries. Czech female students had a significantly higher agreement level for item no. 5 of using child-sized toothbrushes (94.4% vs. 77.8%) and a lower agreement level for item no. 14 of preventing periodontal disease with brushing alone (12% vs. 24.4%) than Czech males. Slovak female students had a significantly higher agreement level for item no. 16 of using plaque-disclosing agents (40.1% vs. 27.1%) than Slovak males (Table 4).

#### 3.3.3. Clinical Experience

On comparing the HU-DBI responses based on clinical experience, clinical students had significantly higher agreement levels for items no. 11 of toothbrushing without toothpaste (63.4% vs. 46.1%), no. 16 of plaque-disclosing agents use (55.6% vs. 43.2%), and no. 20 of positive feedback of treating dentist (84.3% vs. 76.8%) than their preclinical peers in both countries. Contrarily, clinical students had a significantly lower agreement level for item no. 17 of using toothbrushes with hard bristles (4.6% vs. 9.6%) than preclinical students. Additionally, clinical students had a significantly higher agreement level for item no. 5 of using child-sized toothbrushes (7.1% vs. 1.1%) than preclinical students in Slovakia only. (Table 4)

#### 3.3.4. Tobacco Smoking

In both countries, the students who reported smoking tobacco at least once a week had a significantly lower agreement level for item no. 5 of using child-sized toothbrushes (22.1% vs. 35.8%) and higher agreement levels for items no. 14 of preventing periodontal disease through toothbrushing alone (42.6% vs. 25.1%), and no. 15 of delaying dental visits (14.7% vs. 6.4%) than non-smoking students (Table 4).

### 3.4. HU-DBI Scores

The mean HU-DBI score of the entire sample was 8.18 ± 1.80, with Czech students (9.34 ± 1.29) having a significantly higher score (*Sig*. < 0.001) than Slovak students (7.56 ± 1.73). Czech students had significantly higher knowledge (4.35 vs. 3.55) and attitudes scores (2.66 vs. 1.73) than their Slovak counterparts. The gender-based differences were not statistically significant (*Sig*. = 0.316); nevertheless, females exhibited slightly higher scores (Table 5).

**Figure 2:**
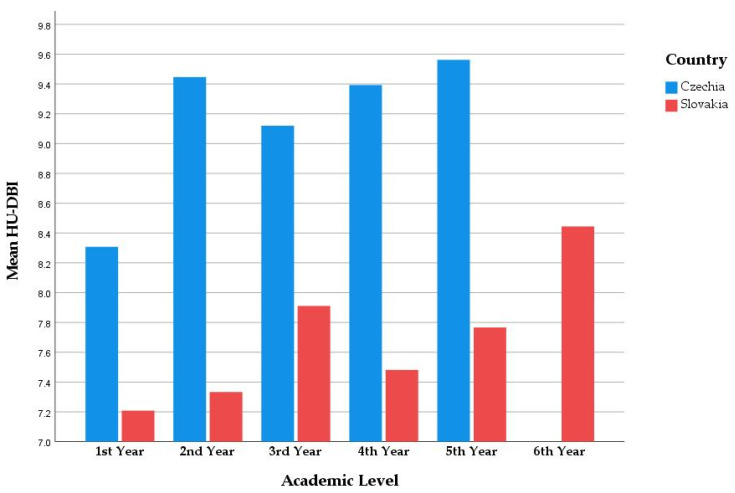
HU-DBI score of Czech and Slovak dental students stratified by state and academic level; Autumn 2021 (*n* = 487).

The highest HU-DBI score was recorded by the fifth-year students (8.87 ± 1.73), while the lowest score was recorded by the first-year students (7.38 ± 1.56). Similarly, the highest knowledge (4.15 ± 0.76) and attitude (2.35 ± 0.82) scores were achieved by the fifth-year students, while the lowest knowledge (3.49 ± 0.91) and attitude (1.68 ± 0.76) scores were achieved by the first-year students. The differences between the academic levels were statistically significant (Figure 2).

**Figure 3:**
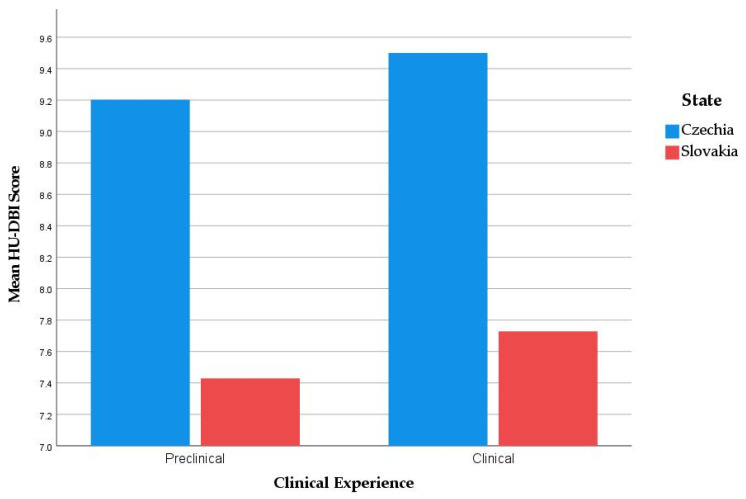
HU-DBI score of Czech and Slovak dental students stratified by state and clinical experience; Autumn 2021 (*n* = 487).

Clinical students from both countries had a significantly higher HU-DBI score (8.35 ± 1.86) than preclinical students (8.04 ± 1.75). The differences were in favour of clinical students in terms of knowledge and attitudes, even though these differences were not statistically significant (Figure 3).

**Figure 4:**
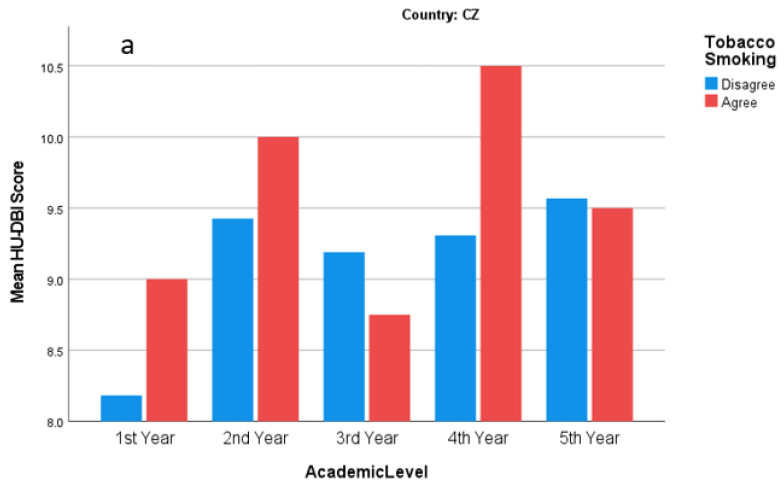

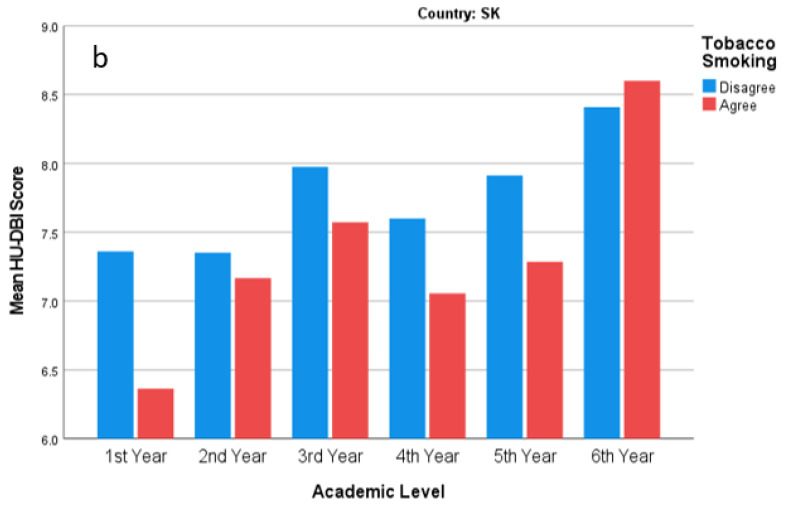
HU-DBI score of (**a**) Czech and (**b**) Slovak dental students stratified by smoking behaviour and academic level; Autumn 2021 (*n* = 487).

The students who reported smoking at least once a week had a significantly lower HU-DBI score (7.63 ± 2.01) than non-smokers (8.27 ± 1.75). Similarly, the students who reported problematic internet use had a significantly lower HU-DBI score (8.11 ± 1.83) than those who did not report it (8.70 ± 1.50). Problematic internet use was associated with lower knowledge (3.79 vs. 4.11) and attitude (2.02 vs. 2.33) scores. Regular dental check-ups were significantly associated with higher HU-DBI (8.23 vs. 7.42) and behaviours (2.33 vs. 1.90) scores. Knowledge and behaviours scores were also higher among the students who reported regular dental check-ups without statistical significance (Figure 4).

#### 3.4.1. Czech Students

In the Czech Republic, gender-based differences were not statistically significant; nevertheless, females scored slightly better. The fifth-year students had the highest HU-DBI score (9.56 ± 1.29), while the first-year students had the lowest HU-DBI score (8.31 ± 1.55). Clinical students (9.50 ± 1.22) and the students who reported regular dental check-ups (9.39 ± 1.23) had higher HU-DBI scores than preclinical students (9.20 ± 1.34) and those who did not report regular dental check-ups (8.62 ± 1.76). HU-DBI scores of the students who reported tobacco smoking and alcohol drinking were not significantly different from their counterparts (Table 6).

#### 3.4.2. Slovak Students

In Slovakia, gender-based differences were not statistically significant. The sixth-year students had the highest HU-DBI score (8.44 ± 1.22), while the first-year students had the lowest HU-DBI score (7.21 ± 1.51). Clinical students (7.73 ± 1.85) and the students who reported regular dental check-ups (7.62 ± 1.71) had significantly higher HU-DBI scores than preclinical students (7.43 ± 1.62) and those who did not report regular dental check-ups (6.56 ± 1.79). HU-DBI scores of the students who reported tobacco smoking, alcohol drinking, and problematic internet use were lower than their counterparts (Table 7).

### 3.5. Year-Over-Year Analysis

#### 3.5.1. Czech Students

The year-over-year (YOY) analysis for Czech students’ HU-DBI scores revealed that the differences between first vs. second year were statistically significant for the knowledge score (*Sig*. = 0.042), attitudes score (*Sig*. = 0.002), and overall HU-DBI score (*Sig*. = 0.007). Additionally, the attitudes score significantly increased from each year to the following one; first vs. second year (*Sig*. = 0.002), second vs. third year (*Sig*. = 0.014), and third vs. fourth year (*Sig*. = 0.033). There were no other significant differences found between the consecutive academic years in terms of HU-DBI scores (Table 8).

#### 3.5.2. Slovak Students

The year-over-year (YOY) analysis for Slovak students’ HU-DBI scores revealed no significant differences between the consecutive academic years in terms of HU-DBI scores. Nevertheless, the largest differences were found between second vs. third year without statistical significance (Table 9).

### 3.6. Regression Analysis of State

According to the univariate analysis for HU-DBI core items, items no. 2 (bleeding gingiva), no. 4 (noticing dental plaque), no. 6 (incapacity to maintain oral health in older age), no. 8 (perceived-efficacy of oral hygiene), no. 10 (receiving professional oral hygiene training), no. 11 (toothbrushing without toothpaste), no. 12 (post-brushing checking), no. 14 (preventing periodontal disease through brushing alone), no.16 (plaque-disclosing agents use), and no. 19 (spending too much time while brushing) were used in the binary logistic regression analysis to predict group membership “country” of the participants. In addition, tobacco smoking and problematic internet use were found significantly associated with students’ country; therefore, they were suggested to be used in the regression model (Table 10).

The suggested model managed to predict the country of the participating students with 80.9% of accuracy. Nagelkerke pseudo R^2^ indicated that the model could explain 52.7% of the variability in the dependent variable (country) (Table 11).

### 3.7. Regression Analysis of Tobacco Smoking

According to the univariate analysis for HU-DBI core items, items no. 14 (preventing periodontal disease through brushing alone) and no. 15 (delaying dental visits) were used in the binary logistic regression analysis to predict group membership “tobacco smoking” of the participants. In addition, Slovak nationality, male gender, and alcohol drinking were found significantly associated with students’ smoking behaviour; therefore, they were suggested to be used in the regression model (Table 12).

The suggested model managed to predict the country of the participating students with 85.6% of accuracy. Nagelkerke pseudo R^2^ indicated that the model could explain 13.7% of the variability in the dependent variable (tobacco smoking) (Table 13).

## 4. Discussion

The present study found that the mean HU-DBI score of Czech dental students (9.34 ± 1.29) was significantly higher than the mean score of Slovak students (7.56 ± 1.73). While the knowledge score (4.35 vs. 3.55) and attitudes score (2.66 vs. 1.73) were significantly higher among Czech students, the behaviours score (2.33 vs. 2.28) was not significantly different between Czech vs. Slovak students. In both countries, female dental students (8.24 ± 1.76) had higher HU-DBI scores than their male colleagues (8.00 ± 1.93); nevertheless, the gender-based differences were not statistically significant. Preclinical students (8.04 vs. 8.35), the students who reported tobacco smoking (7.63 vs. 8.27), and those who reported problematic internet use (8.11 vs. 8.70) had significantly lower HU-DBI scores than their counterparts. On comparing our findings to the HU-DBI-based studies of European dental students, Czech and Slovak students had HU-DBI score (8.18 ± 1.80), which was comparable with the students from Western Europe and Nordic countries, e.g., Swiss (8.02 ± 1.27), Dutch (8.0 ± 1.19), Portuguese (7.74 ± 1.40), Brits (7.33), and Finns (7.15 ± 1.13) students [19,54,55,56]. Our participants’ score was significantly higher than the score of the students from Eastern Europe, e.g., Serbian (6.27 ± 0.27), Lithuanian (6.35 ± 1.43), Croatian (6.62 ± 1.54), and Romanian (6.96) students [57,58,59,60].

While twice-daily brushing with fluoride toothpaste is a universal recommendation for oral hygiene, multiple systematic reviews and meta-analyses revealed that toothpaste has no contribution to the mechanical removal of dental plaque [61,62,63]. Sälzer et al. 2020 confirmed that a reduction in plaque scores by 50% can be achieved by toothbrushing either with or without toothpaste [63]. Therefore, agreement with item no. 11 of toothbrushing without toothpaste and disagreement with item no. 14 of preventing periodontal disease by toothbrushing solely were depicted as indicators for excellent oral health-related awareness and attitudes. Our study found that Czech students were significantly more agreeable with item no. 11 (84.1% vs. 37.5%) and disagreeable with item no. 14 (84.7% vs. 65.9%) than their Slovak counterparts; nevertheless, final-year students had significantly higher agreement levels with item no. 11 than their first-year colleagues in both the Czech Republic (91.7% vs. 53.8%) and Slovakia (66.7% vs. 19.4%). Similar positive trend was previously reported in Romania (freshers: 26% vs. seniors: 58%), Poland (1.9% vs. 33.9%), Greece (11% vs. 64%), Japan (59% vs. 96%), South Korea (3% vs. 88%) [18,60,64,65]. In Croatia, final-year dental students (42.6%) reported using plaque-disclosing agents significantly more than their first-year colleagues (16.1%) [66]. On the other hand, Croatian nurses with completed secondary school (16.3%) and nurses with bachelor’s or master’s degrees (19.6%) did not have significant differences (*Sig*. = 0.671) in terms of plaque-disclosing agents use; thus, indicating the positive impact of dental curricula on dental students’ oral health attitudes [67].

Plaque-disclosing agents use (item no. 16) indicates positive oral health behaviours; therefore, it was incorporated in the HU-DBI scoring system. Recent studies revealed that the vast majority of dental hygienists in the Czech Republic (88.2%) recommend their patients use plaque detectors at home in the form of tablets (78.3%) and mouthwashes (9.9%) due to the ease of their application; nevertheless, more than half of Czech adults reported that they had never visited a dental hygienist in their life [68,69]. The use of plaque-disclosing agents was significantly (*Sig*. < 0.001) higher among Czech (70%) students than their Slovak counterparts (37.2%), even though there was a significant and steady increase (+30%) of their use from the first year to the final year in both the Czech Republic (53.8% vs. 83.3%) and Slovakia (33.3% vs. 63%). In Turkey, several HU-DBI-based studies reported the same increasing pattern of plaque-disclosing agents use from the first year to the final year [70,71,72,73]. On comparing dental students to other healthcare students, e.g., general medicine and nursing students, the use of plaque-disclosing agents was significantly increasing through dental education, while it did not differ between freshers and seniors of other healthcare programs [73,74]. Therefore, it is evident that dental curricula, through their preventive elements, can help in increasing the use of plaque-disclosing agents.

The use of toothbrushes with hard bristles (item no. 17) can be associated with hard dental tissues loss and soft tissues injuries. A randomized controlled trial by Zimmer et al. 2011 revealed that hard bristles had higher efficiency for plaque removal; however, they also caused soft tissue injuries more frequently compared with soft bristles [75]. Other studies concluded that hard dental tissue loss (erosion) had been mediated by stiffness of toothbrushes bristles, and it was mainly caused by toothpaste and their chemical composition [76]. A recent population-based study from Brazil found that bristles stiffness was significantly associated with erosive tooth wear among adolescents [77]. In our study, Slovak students had significantly (*Sig*. < 0.001) higher agreement with item no. 17 of using toothbrushes with hard bristles than their Czech counterparts (10.4% vs. 1.8%, respectively); and in both countries first-year students had significantly higher agreement levels compared with their final-year colleagues. In agreement with our findings, a recent survey for oral health practices of medical and dental hygiene students at the Third Faculty of Medicine, Charles University (Prague, Czech Republic) reported that the vast majority of participating students were using either extra-soft or ultra-soft toothbrushes [78]. Nevertheless, population-based studies for oral hygiene behaviours of Czech adults are recommended to address bristles stiffness for a better understanding of consumption patterns.

Aggressive toothbrushing refers to applying excessive mechanical forces during brushing that may cause tooth surface abrasion [79,80,81,82,83]. Several studies recommended that the application of appropriate mechanical forces during toothbrushing should be an integral part of oral hygiene education in order to avoid the negative consequences of aggressive toothbrushing [79,82]. In our study, Slovak students had a significantly (*Sig*. < 0.001) higher rate of reported aggressive toothbrushing (item no. 18) compared with Czech students, 14.5% vs. 3.5%, respectively. Among Czech students, the rate of aggressive toothbrushing did not differ significantly between preclinical and clinical students, while in Slovakia, first-year students (25%) had a higher rate than final-year students (7.4%). Similar to the Slovak trend, final-year dental students had significantly lower levels of aggressive toothbrushing than their first-year colleagues in Poland (0% vs. 13%), Greece (7% vs. 33%), and Japan (13% vs. 48%) [18,56,64].

Worrying about teeth colour (item no. 3) was one of the few items that were not significantly different between Czech vs. Slovak students or preclinical vs. clinical students, even though this issue was significantly more common among female students than their male peers in both the Czech Republic (97.6% vs. 84.4%) and Slovakia (97.6% vs. 91.4%). Interestingly enough, the responses to items no. 7 of dissatisfaction with gingival colour and no. 13 of worrying about halitosis were not significantly different across gender or clinical experience. Prior HU-DBI-based studies found that female dental students were more worried about their teeth colour (item no. 3) than male students, e.g., Poland (38.6% vs. 20.4%) and Romania (44% vs. 31%) [60,64]. In Brazil, a descriptive cross-sectional study concluded that female dental students were less satisfied with their smiles than their male peers, and the preclinical students were more interested in having brighter teeth than clinical students [84]. While multiple studies revealed no significant differences between female and male dental students in their skills of teeth shade matching, few studies found that female dental students had superior skills [85,86,87]. Another explanation could be based on the finding that females are more concerned with facial appearance; therefore, they are more sensitive to teeth shape and colour and more inclined to seek esthetic treatments [88,89,90,91,92,93].

Female students represented the majority of our sample (76.4%); thus reflecting the female dominance of the dental profession in both the Czech Republic (64.9%) and Slovakia (61.2%), according to the latest reports of the Czech Dental Chamber (ČSK) and the Slovak Chamber of Dentists (SKZL) [41,94]. According to the Council of European Dentists (CED), countries with well-established public oral healthcare systems, such as Nordic and Eastern European countries, used to have higher shares of female dentists, e.g., Poland (78%), and Finland (69%). Additionally, the recent CED report pointed out the rising trend of female dentists in Europe, which was clearly evident in Western countries such as the United Kingdom, which witnessed a significant increase in female dentists proportion from 34% in 2008 to 45% in 2015 and France (36% vs. 40%) [95]. 

Čepová et al. 2018 found that female adults in Slovakia were significantly more likely to visit dentist/dental hygienist for routine check-ups (59.9% vs. 49.1%), report twice-daily toothbrushing (83.5% vs. 72.3%), and use interdental cleaning devices (62.5% vs. 42.1%) than male adults [96]. Similarly, Samohyl et al. 2021 concluded that avoidance of preventive oral healthcare was significantly more common among male adolescents than females in Slovakia [97]. The Health Behaviour in School-aged Children (HBSC) study found a significant difference between female (71.7%) and male (54.8%) adolescents in Slovakia in terms of twice-daily toothbrushing [98]. In the Czech Republic, the HBSC indicated that 32–38% of male and 21% of female adolescents were not brushing their teeth twice a day, even though there was an observed positive trend towards the twice-daily brushing habit among males between 1994 and 2014 [99]. In our sample, the gender-based differences were not statistically significant in HU-DBI scores and the vast majority of items responses, which is in contrast to what was previously reported about oral health behaviours and awareness of general Czech and Slovak populations [96,97,98,99]. Consequently, one may put forward that dental education can contribute to squeezing or probably closing the gender gaps in oral health attitudes and behaviours, which might be a sound reasoning for population-level interventions that target oral health literacy of the public [100,101,102].

Clinical students had a higher HU-DBI score than pre-clinical students in both countries (8.35 vs. 8.04); nevertheless, this difference was only statistically significant among Slovak (7.73 vs. 7.43; *Sig*. = 0.032) not Czech (9.50 vs. 9.20; *Sig*. = 0.166) students, which could be due to sample size differences. The superiority of clinical students in HU-DBI scores was observed in prior studies, e.g., Lithuania (6.81 vs. 5.96), Romania (7.35 vs. 6.60), and Turkey (7.47 vs. 6.00) [58,60,71]. The standard hypothesis for explaining this difference implies that improvement of oral health KAB is a collateral gain from the professional education on oral diseases and prevention, which is gradually received by dental students [59]. On comparing the undergraduate dental curricula of both countries, the courses of preventive dentistry and dental public health are administered earlier in Czech than Slovak universities. In the Czech Republic, the course of preventive dentistry and dental hygiene (B03033) is administered during the first semester (first year) at Charles University (Prague); and the course of preventive dentistry and cariology (ST1/ZUB01) is also administered during the second semester (first year) at Palacky University (Olomouc) [103,104]. On the other hand, the course of preventive dentistry (J-S-ZL-035) is administered during the sixth semester (third year) at Comenius University (Bratislava), and the course of preventive dentistry (SK/PreZL-ZL/15) is administered during the fifth semester (third year) in Pavol Jozef Šafárik University (Košice) [105,106]. The year-over-year analysis (YOY) indicated that the only significant improvement for HU-DBI score occurred among Czech students was between the first vs. second year (*Sig*. = 0.007); thus, it may be depicted as an immediate effect of preventive courses that were administered during the first year.

The reported prevalence of tobacco smoking in our sample was 14%, which is significantly lower than the prevalence of tobacco smoking in both Czech (31.5%) and Slovak (32.3%) general adult populations [107,108]. Tobacco smoking was more significantly common among male students (24.3%) than females (10.8%), which is in agreement with the current demographics of tobacco use in both the Czech Republic (35.4% vs. 22.6%) and Slovakia (39.2% vs. 23.2%) [109]. Notably, Slovak students were significantly more likely to report tobacco smoking (17%) than their Czech counterparts (8.2%). According to the latest European Tobacco Control Scale (ETCS) report of 2019, the rank of the Czech Republic (23rd) had improved by eight positions since the report of 2016 (31st) due to the fact that the country adopted comprehensive anti-smoking legislations since February 2017 and ratified the WHO FCTC Illicit Trade Protocol [110]. The ECTS report of 2019 also showed that the rank of Slovakia (32nd) had dropped by two positions since the 2016 report (30th) as no progress was made since 2010 in the fight against tobacco [110]. Anti-smoking education was first introduced to undergraduate dental curricula in the Czech Republic twenty years ago [111]. The rationale for this move was based on the prior findings on the underestimation of smoking risks by healthcare professionals, including physicians and dentists, who were not reimbursed for helping their patients quit smoking [111]. In our study, the students who reported smoking tobacco at least once a week had a significantly lower HU-DBI score (8.27 vs. 7.63) and a higher agreement level for item no. 15 of delaying dental visits (14.7% vs. 6.4%) compared with non-smoking students. Our findings suggest that tobacco smoking may be associated with poor oral health KAB among dental students; thus, calling for a re-evaluation of the currently implemented anti-smoking curricula in Czech dental schools.

Mravčík et al. 2019 concluded that although alcohol consumption and heavy episodic drinking levels in the Czech Republic are one of the highest worldwide, there was a recent declining trend for alcohol drinking among adolescents and children [112]. A total of 35.5% of our participants reported drinking alcohol at least once a week, with a significant (*Sig*. < 0.001) difference between males (50.4%) and females (30.9%). Longitudinal analysis for HBSC data of Czech adolescents pointed out this significant decline of alcohol drinking between 1994 and 2014, with an increased vulnerability of male adolescents [113]. The same trend was reported by HBSC in Slovakia with similar gender-based differences [114]. A recent large cross-sectional study for American adults revealed that alcohol consumption, especially heavy drinking, was significantly associated with alterations of oral microbiome that might explain the aetiology of multiple alcohol-related diseases [115]. While alcohol drinking was not associated with poor oral health KAB among our participants, it is still imperative to educate and motivate future dentists to perform screening for alcohol use, especially heavy drinking, among their patients as this can be a life-saving intervention for early detection of oral and oropharyngeal cancers [116].

The vast majority of our participants (88.3%) reported using their smartphones and laptops longer than they planned. Problematic internet use had been consistently found among all age groups of Czech society, while the 12–15-year-old adolescents exhibited the highest level of excessive internet use [117]. In our sample, problematic internet use was significantly associated with a lower oral health-related knowledge score (3.79 vs. 4.11), attitudes score (2.02 vs. 2.33), and HU-DBI score (8.11 vs. 8.70). Recently, a national survey-based study for Korean adolescents revealed that problematic internet use affected sleep quality directly and oral health indirectly [118]. Our results warrant further investigation for the potential association between oral health KAB and problematic internet use, especially among younger age groups.

### 4.1. Strengths

To the best of the authors’ knowledge, this was the first study to evaluate the oral health KAB of dental students in the Czech Republic and Slovakia. The use of HU-DBI as a widely used instrument facilitated international comparison of the Czech and Slovak dental students’ outcomes. Following a rigorous methodology for translation and cross-cultural adaptation of the HU-DBI, especially in producing the Czech version, ensured the validity of the translated versions. The identity of the participants was anonymous in order to limit the Hawthorne’s effect and information bias that is predicted to occur with healthcare professionals and students.

### 4.2. Limitations

The first limitation of the present study is the cross-sectional design that did not allow for real-time evaluation of the year-over-year gains of oral health KAB during dental education. Secondly, cross-country comparison was limited in items no. 1 (dental anxiety) and no. 5 (use of child-sized toothbrushes) due to discrepancies of Czech vs. Slovak translations; nevertheless, gender- and academic-level-based comparisons were possible for each country. Thirdly, there was a lack of information on tobacco smoking and alcohol drinking because the investigators aimed to keep the questionnaire as short as possible in order to ensure a satisfactory response rate. Fourthly, the unequal sample sizes of Czech and Slovak students may have limited the cross-country comparison.

### 4.3. Implications

The findings of this study suggest that early implementation of preventive elements in undergraduate dental curricula may yield better and more sustainable oral health gains for the students. Future research on Czech and Slovak dental curricula need to re-evaluate the current anti-smoking components and their impact on students’ views and attitudes. The potential association between problematic internet use and oral health KAB need further investigation, especially among young adult groups, including future healthcare professionals.

## 5. Conclusions

The present study found that the mean HU-DBI score of Czech and Slovak dental students (8.18 ± 1.80) is comparable with the previously reported scores of dental students in Nordic and Western European countries. Czech students (9.34 ± 1.29) had a significantly higher score than their Slovak counterparts (7.56 ± 1.73). In both countries, preclinical students (8.04 vs. 8.35), the students who reported tobacco smoking (7.63 vs. 8.27), and those who reported problematic internet use (8.11 vs. 8.70) had significantly lower HU-DBI scores than their counterparts. In the Czech Republic, the significant increases in HU-DBI scores occurred after the first academic year when the students received preventive dentistry courses; therefore, one can put forward that early implementation of preventive elements in undergraduate dental curricula may yield better and more sustainable oral health gains for the students. Future research on Czech and Slovak dental curricula need to re-evaluate the current anti-smoking components and their impact on students’ views and attitudes.

## Figures and Tables

**Table 1:** Sociodemographic characteristics of Czech and Slovak dental students’ responding to HU-DBI Survey, Autumn 2021 (*n* = 487).

Variable	Outcome	Czech (*n* = 170)	Slovak (*n* = 317)	Total (*n* = 487)	*Sig*.
Gender	Female	125 (73.5%)	247 (77.9%)	372 (76.4%)	0.277
	Male	45 (26.5%)	70 (22.1%)	115 (23.6%)	
Academic Level	First Year	13 (7.6%)	72 (22.7%)	85 (17.5%)	**<0.001**
	Second Year	56 (32.9%)	60 (18.9%)	116 (23.8%)	**<0.001**
	Third Year	25 (14.7%)	45 (14.2%)	70 (14.4%)	0.878
	Fourth Year	28 (16.5%)	83 (26.2%)	111 (22.8%)	**0.015**
	Fifth Year	48 (28.2%)	30 (9.5%)	78 (16%)	**<0.001**
	Sixth Year	*N/A*	27 (8.5%)	27 (5.5%)	*N/A*
Clinical Experience	Preclinical	94 (55.3%)	177 (55.8%)	271 (55.6%)	0.909
	Clinical	76 (44.7%)	140 (44.2%)	216 (44.4%)	

Chi-squared test (*χ*^2^) had been used with a significance level (*Sig*.) ≤ 0.05. The significant values are in **bold** font.

**Table 2:** Health-related behaviours of Czech and Slovak dental students’ responding to HU-DBI survey, Autumn 2021 (*n* = 487).

Variable	Outcome	Czech (*n* = 170)	Slovak (*n* = 317)	*Sig*.	Female (*n* = 372)	Male (*n* = 115)	*Sig*.	Preclinical (*n* = 271)	Clinical (*n* = 216)	*Sig*.	Total (*n* = 487)
Tobacco Smoking	Yes	14 (8.2%)	54 (17%)	**0.008**	40 (10.8%)	28 (24.3%)	**<0.001**	32 (11.8%)	36 (16.7%)	0.124	68 (14%)
	No	156 (91.8%)	263 (83%)		332 (89.2%)	87 (75.7%)		239 (88.2%)	180 (83.3%)		419 (86%)
Alcohol Drinking	Yes	60 (35.3%)	113 (35.6%)	0.938	115 (30.9%)	58 (50.4%)	**<0.001**	92 (33.9%)	81 (37.5%)	0.416	173 (35.5%)
	No	110 (64.7%)	204 (64.4%)		257 (69.1%)	57 (49.6%)		179 (66.1%)	135 (62.5%)		314 (64.5%)
Problematic Internet Use	Yes	142 (83.5%)	288 (90.9%)	**0.017**	332 (89.2%)	98 (85.2%)	0.240	240 (88.6%)	190 (88%)	0.838	430 (88.3%)
	No	28 (16.5%)	29 (9.1%)		40 (10.8%)	17 (14.8%)		31 (11.4%)	26 (12%)		57 (11.7%)
Regular Dental Check-up	Yes	157 (92.4%)	299 (94.3%)	0.396	351 (94.4%)	105 (91.3%)	0.242	257 (94.8%)	199 (92.1%)	0.225	456 (93.6%)
	No	13 (7.6%)	18 (5.7%)		21 (5.6%)	10 (8.7%)		14 (5.2%)	17 (7.9%)		31 (6.4%)

Chi-squared test (*χ*^2^) had been used with a significance level (*Sig*.) ≤ 0.05. The significant values are in **bold** font.

**Table 3:** Czech and Slovak dental students’ responses to HU-DBI items, stratified by academic level, Autumn 2021 (*n* = 487).

Item	Response	State	1st Year (*n* = 85)	2nd Year (*n* = 116)	3rd Year (*n* = 70)	4th Year (*n* = 111)	5th Year (*n* = 78)	6th Year (*n* = 111)	*Sig*. *^U^*	Total (*n* = 487)	*Sig*. ^χ^
No. 1	Agree	CZ	9 (69.2%)	51 (91.1%)	25 (100%)	25 (89.3%)	45 (93.8%)	*N/A*	0.015	155 (91.2%)	**<0.001**
		SK	10 (13.9%)	5 (8.3%)	4 (8.9%)	14 (16.9%)	4 (13.3%)	2 (7.4%)	0.381	39 (12.3%)	
No. 2	Disagree	CZ	12 (92.3%)	56 (100%)	25 (100%)	28 (100%)	48 (100%)	*N/A*	0.055	169 (99.4%)	**<0.001**
		SK	66 (91.7%)	54 (90%)	42 (93.3%)	73 (88%)	28 (93.3%)	26 (96.3%)	0.426	289 (91.2%)	
No. 3	Agree	CZ	13 (100%)	52 (92.9%)	21 (84%)	28 (100%)	46 (95.8%)	*N/A*	0.458	160 (94.1%)	0.288
		SK	69 (95.8%)	58 (96.7%)	42 (93.3%)	81 (97.6%)	28 (93.3%)	27 (100%)	0.284	305 (96.2%)	
No. 4	Agree	CZ	2 (15.4%)	10 (17.9%)	3 (12%)	3 (10.7%)	10 (20.8%)	*N/A*	0.664	28 (16.5%)	**<0.001**
		SK	26 (36.1%)	15 (25%)	16 (35.6%)	29 (34.9%)	9 (30%)	6 (22.2%)	0.190	101 (31.9%)	
No. 5	Agree	CZ	11 (84.6%)	48 (85.7%)	24 (96%)	26 (92.9%)	44 (91.7%)	*N/A*	0.453	153 (90%)	**<0.001**
		SK	1 (1.4%)	0 (0%)	1 (2.2%)	7 (8.4%)	1 (3.3%)	2 (7.4%)	0.122	12 (3.8%)	
No. 6	Disagree	CZ	11 (84.6%)	54 (96.4%)	24 (96%)	28 (100%)	48 (100%)	*N/A*	**0.006**	165 (97.1%)	**<0.001**
		SK	51 (70.8%)	41 (68.3%)	35 (77.8%)	56 (67.5%)	18 (60%)	20 (74.1%)	0.751	221 (69.7%)	
No. 7	Agree	CZ	1 (7.7%)	1 (1.8%)	1 (4%)	0 (0%)	1 (2.1%)	*N/A*	0.318	4 (2.4%)	**0.001**
		SK	6 (8.3%)	9 (15%)	7 (15.6%)	7 (8.4%)	2 (6.7%)	2 (7.4%)	0.881	33 (10.4%)	
No. 8	Disagree	CZ	12 (92.3%)	52 (92.9%)	23 (92%)	26 (92.9%)	46 (95.8%)	*N/A*	0.605	159 (93.9%)	**<0.001**
		SK	54 (75%)	47 (78.3%)	37 (82.2%)	64 (77.1%)	26 (86.7%)	25 (92.6%)	**0.053**	253 (79.8%)	
No. 9	Agree	CZ	9 (69.2%)	44 (78.6%)	20 (80%)	20 (71.4%)	38 (79.2%)	*N/A*	0.454	131 (77.1%)	0.428
		SK	49 (68.1%)	50 (83.3%)	39 (86.7%)	69 (83.1%)	22 (73.3%)	25 (92.6%)	**0.013**	254 (80.1%)	
No. 10	Disagree	CZ	10 (76.9%)	54 (96.4%)	23 (92%)	27 (96.4%)	46 (95.8%)	*N/A*	**0.029**	160 (94.1%)	**<0.001**
		SK	52 (72.2%)	43 (71.7%)	34 (75.6%)	59 (71.1%)	26 (86.7%)	22 (81.5%)	0.347	236 (74.4%)	
No. 11	Agree	CZ	7 (53.8%)	50 (89.3%)	19 (76%)	23 (82.1%)	44 (91.7%)	*N/A*	**0.001**	143 (84.1%)	**<0.001**
		SK	14 (19.4%)	18 (30%)	17 (37.8%)	37 (44.6%)	15 (50%)	18 (66.7%)	**<0.001**	119 (37.5%)	
No. 12	Agree	CZ	12 (92.3%)	44 (78.6%)	16 (64%)	17 (60.7%)	29 (60.4%)	*N/A*	**0.031**	118 (69.4%)	**0.016**
		SK	58 (80.6%)	49 (81.7%)	35 (77.8%)	67 (80.7%)	21 (70%)	21 (77.8%)	0.760	251 (79.2%)	
No. 13	Agree	CZ	6 (46.2%)	20 (35.7%)	10 (40%)	10 (35.7%)	16 (33.3%)	*N/A*	0.397	62 (36.5%)	0.567
		SK	17 (23.6%)	21 (35%)	26 (57.8%)	27 (32.5%)	18 (60%)	15 (55.6%)	**0.003**	124 (39.1%)	
No. 14	Disagree	CZ	10 (76.9%)	51 (91.1%)	18 (72%)	27 (96.4%)	38 (79.2%)	*N/A*	0.862	144 (84.7%)	**<0.001**
		SK	50 (69.4%)	45 (75%)	29 (64.4%)	49 (59%)	20 (66.7%)	16 (59.3%)	0.341	209 (65.9%)	
No. 15	Disagree	CZ	12 (92.3%)	54 (96.4%)	24 (96%)	27 (96.4%)	44 (91.7%)	*N/A*	0.941	161 (94.7%)	0.160
		SK	65 (90.3%)	54 (90%)	44 (97.8%)	75 (90.4%)	25 (83.3%)	26 (96.3%)	0.330	289 (91.2%)	
No. 16	Agree	CZ	7 (53.8%)	34 (60.7%)	18 (72%)	20 (71.4%)	40 (83.3%)	*N/A*	**0.026**	119 (70%)	**<0.001**
		SK	24 (33.3%)	16 (26.7%)	18 (40%)	27 (32.5%)	16 (53.3%)	17 (63%)	**0.008**	118 (37.2%)	
No. 17	Agree	CZ	2 (15.4%)	0 (0%)	1 (4%)	0 (0%)	0 (0%)	*N/A*	**0.006**	3 (1.8%)	**<0.001**
		SK	13 (18.1%)	7 (11.7%)	3 (6.7%)	9 (10.8%)	1 (3.3%)	0 (0%)	**0.018**	33 (10.4%)	
No. 18	Agree	CZ	0 (0%)	2 (3.6%)	0 (0%)	2 (7.1%)	2 (4.2%)	*N/A*	0.458	6 (3.5%)	**<0.001**
		SK	18 (25%)	8 (13.3%)	2 (4.4%)	11 (13.3%)	5 (16.7%)	2 (7.4%)	**0.053**	46 (14.5%)	
No. 19	Agree	CZ	4 (30.8%)	26 (46.4%)	15 (60%)	17 (60.7%)	28 (58.3%)	*N/A*	0.080	90 (52.9%)	**<0.001**
		SK	10 (13.9%)	8 (13.3%)	10 (22.2%)	16 (19.3%)	7 (23.3%)	6 (22.2%)	0.318	57 (18%)	
No. 20	Agree	CZ	9 (69.2%)	43 (76.8%)	22 (88%)	26 (92.9%)	43 (89.6%)	*N/A*	0.069	143 (84.1%)	0.102
		SK	53 (73.6%)	46 (76.7%)	35 (77.8%)	66 (79.5%)	23 (76.7%)	24 (88.9%)	0.105	247 (77.9%)	

*^U^* Mann–Whitney test (*U*) between first- vs. final-year students had been used with a significance level (*Sig*.) ≤ 0.05. *^χ^* Chi-squared test (*χ*^2^) between Czech vs. Slovak students had been used with a significance level (*Sig*.) ≤ 0.05. The significant values are in **bold** font.

**Table 4:** Czech and Slovak dental students’ responses to HU-DBI items, stratified by gender, clinical experience and tobacco smoking, Autumn 2021 (*n* = 487).

Item	Response	State	Female (*n* = 372)	Male (*n* = 115)	*Sig*.	Preclinical (*n* = 271)	Clinical (*n* = 216)	*Sig*.	Non-Smoker (*n* = 419)	Smoker (*n* = 68)	*Sig*.
No. 1	Agree	CZ	116 (92.8%)	39 (86.7%)	0.228 *	85 (90.4%)	70 (92.1%)	0.701	145 (92.9%)	10 (71.4%)	0.023 *
		SK	31 (12.6%)	8 (11.4%)	0.801	19 (10.7%)	20 (14.3%)	0.339	29 (11%)	10 (18.5%)	0.127
		Total	147 (39.5%)	47 (40.9%)	0.796	104 (38.4%)	90 (41.7%)	0.461	174 (41.5%)	20 (29.4%)	0.058
No. 2	Disagree	CZ	124 (99.2%)	45 (100%)	1.000 *	93 (98.9%)	76 (100%)	1.000 *	155 (99.4%)	14 (100%)	1.000 *
		SK	226 (91.5%)	63 (90%)	0.697	162 (91.5%)	127 (90.7%)	0.800	240 (91.3%)	49 (90.7%)	1.000 *
		Total	350 (94.1%)	108 (93.9%)	0.945	255 (94.1%)	203 (94%)	0.958	395 (94.3%)	63 (92.6%)	0.581 *
No. 3	Agree	CZ	122 (97.6%)	38 (84.4%)	0.004 *	86 (91.5%)	74 (97.4%)	0.188 *	146 (93.6%)	14 (100%)	1.000 *
		SK	241 (97.6%)	64 (91.4%)	0.028 *	169 (95.5%)	136 (97.1%)	0.441	251 (95.4%)	54 (100%)	0.231 *
		Total	363 (97.6%)	102 (88.7%)	**<0.001**	255 (94.1%)	210 (97.2%)	0.099	397 (94.7%)	68 (100%)	0.057 *
No. 4	Agree	CZ	19 (15.2%)	9 (20%)	0.457	15 (16%)	13 (17.1%)	0.841	24 (15.4%)	4 (28.6%)	0.252 *
		SK	77 (31.2%)	24 (34.3%)	0.622	57 (32.2%)	44 (31.4%)	0.883	88 (33.5%)	13 (24.1%)	0.178
		Total	96 (25.8%)	33 (28.7%)	0.539	72 (26.6%)	57 (26.4%)	0.964	112 (26.7%)	17 (25%)	0.764
No. 5	Agree	CZ	118 (94.4%)	35 (77.8%)	0.003 *	83 (88.3%)	70 (92.1%)	0.411	140 (89.7%)	13 (92.9%)	1.000 *
		SK	8 (3.2%)	4 (5.7%)	0.308 *	2 (1.1%)	10 (7.1%)	**0.005**	10 (3.8%)	2 (3.7%)	1.000 *
		Total	126 (33.9%)	39 (33.9%)	0.993	85 (31.4%)	80 (37%)	0.189	150 (35.8%)	15 (22.1%)	**0.026**
No. 6	Disagree	CZ	122 (97.6%)	43 (95.6%)	0.609 *	89 (94.7%)	76 (100%)	0.066 *	151 (96.8%)	14 (100%)	1.000 *
		SK	177 (71.7%)	44 (62.9%)	0.157	127 (71.8%)	94 (67.1%)	0.375	184 (70%)	37 (68.5%)	0.833
		Total	299 (80.4%)	87 (75.7%)	0.275	216 (79.7%)	170 (78.7%)	0.787	335 (80%)	51 (75%)	0.350
No. 7	Agree	CZ	2 (1.2%)	2 (4.4%)	0.286 *	3 (3.2%)	1 (1.3%)	0.629 *	3 (1.9%)	1 (7.1%)	0.217 *
		SK	25 (10.1%)	8 (11.4%)	0.752	22 (12.4%)	11 (7.9%)	0.186	28 (10.6%)	5 (9.3%)	0.761
		Total	27 (7.3%)	10 (8.7%)	0.611	25 (9.2%)	12 (5.6%)	0.129	31 (7.4%)	6 (8.8%)	0.681
No. 8	Disagree	CZ	117 (93.6%)	42 (93.3%)	0.950 *	87 (92.6%)	72 (94.7%)	0.756 *	146 (93.6%)	13 (92.9%)	1.000 *
		SK	195 (78.9%)	58 (82.9%)	0.472	138 (78%)	115 (82.1%)	0.358	211 (80.2%)	42 (77.8%)	0.683
		Total	312 (83.9%)	100 (87%)	0.423	225 (83%)	187 (86.6%)	0.281	357 (85.2%)	55 (80.9%)	0.360
No. 9	Agree	CZ	98 (78.4%)	33 (73.3%)	0.488	73 (77.7%)	58 (76.3%)	0.836	121 (77.6%)	10 (71.4%)	0.740 *
		SK	199 (80.6%)	55 (78.6%)	0.712	138 (78%)	116 (82.9%)	0.279	213 (81%)	41 (75.9%)	0.396
		Total	297 (79.8%)	88 (76.5%)	0.445	211 (77.9%)	174 (80.6%)	0.468	334 (79.7%)	51 (75%)	0.376
No. 10	Disagree	CZ	120 (96%)	40 (88.9%)	0.132 *	87 (92.6%)	73 (96.1%)	0.515 *	147 (94.2%)	13 (92.9%)	0.587 *
		SK	186 (75.3%)	50 (71.4%)	0.512	129 (72.9%)	107 (76.4%)	0.472	196 (74.5%)	40 (74.1%)	0.945
		Total	306 (82.3%)	90 (78.3%)	0.336	216 (79.7%)	180 (83.3%)	0.307	343 (81.9%)	53 (77.9%)	0.442
No. 11	Agree	CZ	106 (84.8%)	37 (82.2%)	0.685	76 (80.9%)	67 (88.2%)	0.195	132 (84.6%)	11 (78.6%)	0.469 *
		SK	94 (38.1%)	25 (35.7%)	0.721	49 (27.7%)	70 (50%)	**<0.001**	95 (36.1%)	24 (44.4%)	0.250
		Total	200 (53.8%)	62 (53.9%)	0.978	125 (46.1%)	137 (63.4%)	**<0.001**	227 (54.2%)	35 (51.5%)	0.678
No. 12	Agree	CZ	90 (72%)	28 (62.2%)	0.222	72 (76.6%)	46 (60.5%)	**0.024**	108 (69.2%)	10 (71.4%)	1.000 *
		SK	198 (80.2%)	53 (75.7%)	0.419	142 (80.2%)	109 (77.9%)	0.606	212 (80.6%)	39 (72.2%)	0.167
		Total	288 (77.4%)	81 (70.4%)	0.127	214 (79%)	155 (71.8%)	0.065	320 (76.4%)	49 (72.1%)	0.441
No. 13	Agree	CZ	43 (34.4%)	19 (42.2%)	0.350	36 (38.3%)	26 (34.2%)	0.582	55 (35.3%)	7 (50%)	0.272
		SK	94 (38.1%)	30 (42.9%)	0.468	64 (36.2%)	60 (42.9%)	0.225	99 (37.6%)	25 (46.3%)	0.235
		Total	137 (36.8%)	49 (42.6%)	0.265	100 (36.9%)	86 (39.8%)	0.511	154 (36.8%)	32 (47.1%)	0.105
No. 14	Disagree	CZ	110 (88%)	34 (75.6%)	**0.047**	79 (84%)	65 (85.5%)	0.789	133 (85.3%)	11 (78.6%)	0.452 *
		SK	163 (66%)	46 (65.7%)	0.965	124 (70.1%)	85 (60.7%)	0.081	181 (68.8%)	28 (51.9%)	**0.017**
		Total	273 (73.4%)	80 (69.6%)	0.423	203 (74.9%)	150 (69.4%)	0.180	314 (74.9%)	39 (57.4%)	**0.003**
No. 15	Disagree	CZ	117 (93.6%)	44 (97.8%)	0.448 *	90 (95.7%)	71 (93.4%)	0.515 *	148 (94.9%)	13 (92.9%)	0.548 *
		SK	229 (92.7%)	60 (85.7%)	0.069	163 (92.1%)	126 (90%)	0.515	244 (92.8%)	45 (83.3%)	0.035 *
		Total	346 (93%)	104 (90.4%)	0.362	253 (93.4%)	197 (91.2%)	0.373	392 (93.6%)	58 (85.3%)	**0.017**
No. 16	Agree	CZ	87 (69.6%)	32 (71.1%)	0.850	59 (62.8%)	60 (78.9%)	**0.022**	109 (69.9%)	10 (71.4%)	1.000 *
		SK	99 (40.1%)	19 (27.1%)	**0.048**	58 (32.8%)	60 (42.9%)	0.065	97 (36.9%)	21 (38.9%)	0.781
		Total	186 (50%)	51 (44.3%)	0.289	117 (43.2%)	120 (55.6%)	**0.007**	206 (49.2%)	31 (45.6%)	0.584
No. 17	Agree	CZ	1 (0.8%)	2 (4.4%)	0.171 *	3 (3.2%)	0 (0%)	0.254	3 (1.9%)	0 (0%)	1.000 *
		SK	24 (9.7%)	9 (12.9%)	0.448	23 (13%)	10 (7.1%)	0.090	31 (11.8%)	2 (3.7%)	0.076
		Total	25 (6.7%)	11 (9.6%)	0.308	26 (9.6%)	10 (4.6%)	**0.038**	34 (8.1%)	2 (2.9%)	0.130
No. 18	Agree	CZ	3 (2.4%)	3 (6.7%)	0.190 *	2 (2.1%)	4 (5.3%)	0.409	6 (3.8%)	0 (0%)	1.000 *
		SK	34 (13.8%)	12 (17.1%)	0.479	28 (15.8%)	18 (12.9%)	0.457	38 (14.4%)	8 (14.8%)	0.945
		Total	37 (9.9%)	15 (13%)	0.347	30 (11.1%)	22 (10.2%)	0.753	44 (10.5%)	8 (11.8%)	0.754
No. 19	Agree	CZ	68 (54.4%)	22 (48.9%)	0.525 *	45 (47.9%)	45 (59.2%)	0.141	81 (51.9%)	9 (64.3%)	0.375
		SK	43 (17.4%)	14 (20%)	0.618	28 (15.8%)	29 (20.7%)	0.260	49 (18.6%)	8 (14.8%)	0.506
		Total	111 (29.8%)	36 (31.3%)	0.765	73 (26.9%)	74 (34.3%)	0.080	130 (31%)	17 (25%)	0.315
No. 20	Agree	CZ	106 (84.8%)	37 (82.2%)	0.685	74 (78.7%)	69 (90.8%)	**0.032**	132 (84.6%)	11 (78.6%)	0.469 *
		SK	193 (78.1%)	54 (77.1%)	0.859	134 (75.7%)	113 (80.7%)	0.286	206 (78.3%)	41 (75.9%)	0.698
		Total	299 (80.4%)	91 (79.1%)	0.770	208 (76.8%)	182 (84.3%)	**0.039**	338 (80.7%)	52 (76.5%)	0.421

Chi-squared test (*χ*^2^) and Fisher’s exact test (*) had been used with a significance level (*Sig*.) ≤ 0.05. The significant values are in **bold** font.

**Table 5:** HU-DBI scores of Czech and Slovak dental students, Autumn 2021 (*n* = 487).

Variable	Outcome	Knowledge (0–5)	*Sig.*	Attitudes (0–3)	*Sig.*	Behaviours (0–4)	*Sig.*	HU-DBI (0–12)	*Sig.*
State	Czech Republic	4.35 ± 0.65	**<0.001**	2.66 ± 0.56	**<0.001**	2.33 ± 0.83	0.488	9.34 ± 1.29	**<0.001**
	Slovakia	3.55 ± 0.88		1.73 ± 0.85		2.28 ± 0.88		7.56 ± 1.73	
Gender	Female	3.83 ± 0.90	0.772	2.08 ± 0.88	0.376	2.33 ± 0.83	0.185	8.24 ± 1.76	0.316
	Male	3.81 ± 0.87		1.99 ± 0.88		2.20 ± 0.98		8.00 ± 1.93	
Academic Level	First Year	3.49 ± 0.91	**<0.001**	1.68 ± 0.76	**0.002**	2.20 ± 0.95	0.061	7.38 ± 1.56	**<0.001**
	Second Year	3.86 ± 0.85		2.23 ± 0.87		2.26 ± 0.89		8.35 ± 1.87	
	Third Year	3.96 ± 0.79		2.03 ± 0.82		2.36 ± 0.92		8.34 ± 1.53	
	Fourth Year	3.71 ± 1.02		1.98 ± 0.94		2.27 ± 0.82		7.96 ± 1.99	
	Fifth Year	4.15 ± 0.76		2.35 ± 0.82		2.37 ± 0.81		8.87 ± 1.73	
	Sixth Year	3.89 ± 0.58		2.00 ± 0.88		2.56 ± 0.70		8.44 ± 1.22	
Clinical Experience	Preclinical	3.77 ± 0.87	0.070	2.01 ± 0.85	0.097	2.27 ± 0.91	0.301	8.04 ± 1.75	**0.016**
	Clinical	3.89 ± 0.91		2.12 ± 0.91		2.34 ± 0.80		8.35 ± 1.86	
Tobacco Smoking	Yes	3.62 ± 1.02	0.073	1.84 ± 0.89	**0.024**	2.18 ± 0.90	0.292	7.63 ± 2.01	**0.012**
	No	3.86 ± 0.86		2.09 ± 0.87		2.32 ± 0.86		8.27 ± 1.75	
Alcohol Drinking	Yes	3.85 ± 0.89	0.532	2.02 ± 0.88	0.496	2.31 ± 0.88	0.782	8.18 ± 1.87	0.798
	No	3.81 ± 0.89		2.07 ± 0.88		2.29 ± 0.86		8.18 ± 1.77	
Problematic Internet Use	Yes	3.79 ± 0.90	**0.015**	2.02 ± 0.89	**0.016**	2.30 ± 0.86	0.817	8.11 ± 1.83	**0.036**
	No	4.11 ± 0.72		2.33 ± 0.72		2.26 ± 0.92		8.70 ± 1.50	
Regular Dental Check-up	Yes	3.84 ± 0.88	0.163	2.06 ± 0.87	0.556	2.33 ± 0.84	**0.041**	8.23 ± 1.78	**0.016**
	No	3.58 ± 1.06		1.94 ± 1.00		1.90 ± 1.08		7.42 ± 2.03	

Mann–Whitney test (*U*) and Jonckheere-Terpstra test (*JT*) had been used with a significance level (*Sig*.) ≤ 0.05. The significant values are in **bold** font.

**Table 6:** HU-DBI scores of Czech dental students, Autumn 2021 (*n* = 170).

Variable	Outcome	Knowledge (0–5)	*Sig.*	Attitudes (0–3)	*Sig.*	Behaviours (0–4)	*Sig.*	HU-DBI (0–12)	*Sig.*
Gender	Female	4.37 ± 0.67	0.336	2.70 ± 0.52	0.080	2.35 ± 0.81	0.631	9.42 ± 1.23	0.138
	Male	4.29 ± 0.59		2.53 ± 0.63		2.27 ± 0.92		9.09 ± 1.44	
Academic Level	First Year	3.85 ± 0.80	**0.044**	2.15 ± 0.80	0.259	2.31 ± 0.86	0.596	8.31 ± 1.55	0.074
	Second Year	4.32 ± 0.61		2.77 ± 0.47		2.36 ± 0.84		9.45 ± 1.14	
	Third Year	4.40 ± 0.65		2.44 ± 0.65		2.28 ± 1.02		9.12 ± 1.48	
	Fourth Year	4.46 ± 0.64		2.79 ± 0.42		2.14 ± 0.80		9.39 ± 1.10	
	Fifth Year	4.42 ± 0.61		2.71 ± 0.50		2.44 ± 0.74		9.56 ± 1.29	
Clinical Experience	Preclinical	4.28 ± 0.66	0.119	2.60 ± 0.61	0.154	2.33 ± 0.89	0.901	9.20 ± 1.34	0.166
	Clinical	4.43 ± 0.62		2.74 ± 0.47		2.33 ± 0.77		9.50 ± 1.22	
Tobacco Smoking	Yes	4.43 ± 0.65	0.620	2.57 ± 0.65	0.590	2.43 ± 1.09	0.541	9.43 ± 1.40	0.923
	No	4.34 ± 0.65		2.67 ± 0.55		2.32 ± 0.81		9.33 ± 1.29	
Alcohol Drinking	Yes	4.35 ± 0.63	0.972	2.63 ± 0.55	0.538	2.43 ± 0.85	0.212	9.42 ± 1.20	0.786
	No	4.35 ± 0.66		2.67 ± 0.56		2.27 ± 0.82		9.29 ± 1.34	
Problematic Internet Use	Yes	4.35 ± 0.65	0.718	2.68 ± 0.54	0.425	2.33 ± 0.81	0.964	9.36 ± 1.28	0.436
	No	4.32 ± 0.61		2.57 ± 0.63		2.32 ± 0.98		9.21 ± 1.37	
Regular Dental Check-up	Yes	4.36 ± 0.63	0.352	2.66 ± 0.56	0.968	2.38 ± 0.77	0.076	9.39 ± 1.23	0.055
	No	4.15 ± 0.80		2.69 ± 0.48		1.77 ± 1.30		8.62 ± 1.76	

Mann–Whitney test (*U*) and Jonckheere-Terpstra test (*JT*) had been used with a significance level (*Sig*.) ≤ 0.05. The significant values are in **bold** font.

**Table 7:** HU-DBI scores of Slovak dental students, Autumn 2021 (*n* = 317).

Variable	Outcome	Knowledge (0–5)	*Sig.*	Attitudes (0–3)	*Sig.*	Behaviours (0–4)	*Sig.*	HU-DBI (0–12)	*Sig.*
Gender	Female	3.56 ± 0.88	0.646	1.76 ± 0.84	0.392	2.32 ± 0.84	0.174	7.64 ± 1.68	0.248
	Male	3.50 ± 0.88		1.64 ± 0.85		2.16 ± 1.02		7.30 ± 1.88	
Academic Level	First Year	3.43 ± 0.92	**0.028**	1.60 ± 0.73	0.074	2.18 ± 0.97	0.072	7.21 ± 1.51	**0.002**
	Second Year	3.43 ± 0.83		1.73 ± 0.86		2.17 ± 0.92		7.33 ± 1.85	
	Third Year	3.71 ± 0.76		1.80 ± 0.82		2.40 ± 0.86		7.91 ± 1.40	
	Fourth Year	3.46 ± 1.00		1.71 ± 0.92		2.31 ± 0.83		7.48 ± 2.00	
	Fifth Year	3.73 ± 0.79		1.77 ± 0.90		2.27 ± 0.91		7.77 ± 1.79	
	Sixth Year	3.89 ± 0.58		2.00 ± 1.73		2.56 ± 0.70		8.44 ± 1.22	
Clinical Experience	Preclinical	3.50 ± 0.85	0.205	1.69 ± 0.80	0.317	2.23 ± 0.93	0.240	7.43 ± 1.62	**0.032**
	Clinical	3.60 ± 0.90		1.78 ± 0.91		2.35 ± 0.82		7.73 ± 1.85	
Tobacco Smoking	Yes	3.41 ± 1.00	0.303	1.65 ± 0.85	0.394	2.11 ± 0.84	0.167	7.17 ± 1.89	0.113
	No	3.57 ± 0.85		1.75 ± 0.85		2.32 ± 0.89		7.64 ± 1.69	
Alcohol Drinking	Yes	3.58 ± 0.89	0.355	1.70 ± 0.84	0.519	2.25 ± 0.89	0.598	7.53 ± 1.83	0.969
	No	3.52 ± 0.87		1.75 ± 0.85		2.30 ± 0.88		7.58 ± 1.68	
Problematic Internet Use	Yes	3.51 ± 0.88	**0.031**	1.69 ± 0.85	**0.015**	2.29 ± 0.89	0.725	7.50 ± 1.75	**0.033**
	No	3.90 ± 0.77		2.10 ± 0.72		2.21 ± 0.86		8.21 ± 1.47	
Regular Dental Check-up	Yes	3.57 ± 0.86	0.082	1.75 ± 0.84	0.092	2.30 ± 0.88	0.238	7.62 ± 1.71	**0.017**
	No	3.17 ± 1.04		1.39 ± 0.92		2.00 ± 0.91		6.56 ± 1.79	

Mann–Whitney test (*U*) and Jonckheere-Terpstra test (*JT*) had been used with a significance level (*Sig*.) ≤ 0.05. The significant values are in **bold** font.

**Table 8:** Pairwise comparison of Czech dental students’ HU-DBI scores across consecutive academic levels, Autumn 2021 (*n* = 170).

Pair	Knowledge		Attitudes		Behaviours		HU-DBI	
	Mean Rank	*Sig.*	Mean Rank	*Sig.*	Mean Rank	*Sig.*	Mean Rank	*Sig.*
1st Year vs. 2nd Year	25.92/37.11	**0.042**	22.69/37.86	**0.002**	33.96/35.24	0.822	21.88/38.04	**0.007**
2nd Year vs. 3rd Year	40.05/43.12	0.544	44.43/33.32	**0.014**	41.04/40.90	0.978	42.58/37.46	0.350
3rd Year vs. 4th Year	26.22/27.70	0.697	23.04/30.54	**0.033**	28.40/25.75	0.504	25.72/28.14	0.557
4th Year vs. 5th Year	39.66/37.82	0.694	39.96/37.65	0.557	34.09/41.07	0.149	36.23/39.82	0.481

Mann–Whitney (*U*) test was used with a significance level (*Sig.*) ≤ 0.05. The significant values are in **bold** font.

**Table 9:** Pairwise comparison of Slovak dental students’ HU-DBI scores across consecutive academic levels, Autumn 2021 (*n* = 317).

Pair	Knowledge		Attitudes		Behaviours		HU-DBI	
	Mean Rank	*Sig.*	Mean Rank	*Sig.*	Mean Rank	*Sig.*	Mean Rank	*Sig.*
1st Year vs. 2nd Year	66.96/65.95	0.870	64.22/69.23	0.421	66.74/66.22	0.935	65.74/67.42	0.798
2nd Year vs. 3rd Year	49.18/58.10	0.103	51.64/54.81	0.575	49.73/57.37	0.178	48.98/58.36	0.113
3rd Year vs. 4th Year	69.43/61.83	0.229	66.86/63.22	0.575	67.27/63.00	0.506	67.81/62.70	0.450
4th Year vs. 5th Year	54.77/63.17	0.190	56.39/58.70	0.727	57.31/56.13	0.857	55.87/60.13	0.535
5th Year vs. 6th Year	28.05/30.06	0.591	27.17/31.04	0.352	26.45/31.83	0.188	26.38/31.91	0.198

Mann–Whitney (*U*) test was used with a significance level (*Sig.*) ≤ 0.05.

**Table 10:** Predictors of state membership; Autumn 2021 (*n* = 487).

Predictor	Beta	S.E.	Wald	df	AOR	95% CI	*Sig*.
Item No. 2: Disagree	−1.83	1.17	2.45	1	0.161	0.016–1.583	0.117
Item No. 4: Agree	0.20	0.32	0.38	1	1.22	0.655–2.253	0.537
Item No. 6: Disagree	−2.07	0.51	16.40	1	0.13	0.047–0.344	**<0.001**
Item No. 8: Disagree	−0.90	0.43	4.36	1	0.41	0.176–0.947	**0.037**
Item No. 10: Disagree	−0.76	0.44	2.92	1	0.47	0.196–1.118	0.087
Item No. 11: Agree	−1.58	0.28	31.45	1	0.21	0.118–0.357	**<0.001**
Item No. 12: Agree	0.35	0.29	1.41	1	1.42	0.797–2.516	0.236
Item No. 14: Disagree	−0.74	0.31	5.72	1	0.48	0.262–0.876	**0.017**
Item No. 16: Agree	−0.87	0.26	11.21	1	0.42	0.250–0.698	**<0.001**
Item No. 19: Agree	−1.62	0.28	34.32	1	0.20	0.115–0.340	**<0.001**
Tobacco Smoking: Yes	0.89	0.41	4.71	1	2.43	1.090–5.425	**0.030**
Problematic Internet Use: Yes	0.12	0.36	0.10	1	1.12	0.553–2.279	0.748

Logistic regression had been used with a significance level (*Sig*.) ≤ 0.05. The Czech Republic was coded as “0” and Slovakia was coded “1”. All significant associations are in **bold** font.

**Table 11:** Observed and predicted group membership of state; Autumn 2021 (*n* = 487).

Observed Group		Predicted Group		Correct Percentage
		Czech Republic	Slovakia	
State	Czech Republic	118	52	69.4%
	Slovakia	41	276	87.1%
Overall				80.9%

The cut-off value is 0.50. Nagelkerke R^2^ = 0.527.

**Table 12:** Predictors of tobacco smoking among Czech and Slovak dental students; Autumn 2021 (*n* = 487).

Predictor.	Beta	S.E.	Wald	df	AOR	95% CI	*Sig*.
State: Slovakia	0.79	0.33	5.59	1	2.20	1.14–4.21	**0.018**
Gender: Male	0.88	0.29	9.09	1	2.40	1.36–4.24	**0.003**
Alcohol Drinking: Yes	0.83	0.28	9.02	1	2.30	1.34–3.97	**0.003**
Item No. 14: Agree	0.63	0.28	4.91	1	1.87	1.08–3.26	**0.027**
Item No. 15: Agree	0.65	0.43	2.32	1	1.92	0.83–4.42	0.128

Logistic regression had been used with a significance level (*Sig*.) ≤ 0.05. All significant associations are in **bold** font.

**Table 13:** Observed and predicted group membership of tobacco smoking among Czech and Slovak dental students; Autumn 2021 (*n* = 487).

Observed Group		Predicted Group		Correct Percentage
		Non-Smoker	Smoker	
Tobacco Smoking	Non-smoker	416	3	99.3%
	Smoker	67	1	1.5%
Overall				85.6%

The cut-off value is 0.50. Nagelkerke R^2^ = 0.137.

## Data Availability

The data that support the findings of this study are available from the corresponding author upon reasonable request.

## Supplementary Materials

The following supporting information can be downloaded at: https://www.mdpi.com/article/10.3390/ijerph19052717/s1, Table S1. Test-re-test Reliability of HU-DBI Czech Version.

## Appendix A

**Table A1:** Hiroshima University Dental Behavioural Inventory (HU-DBI) in English (EN), Czech (CZ), and Slovak (SK).

#	Language	Question Otázka Otázka	Agree Souhlasím Súhlasím	Disagree Nesouhlasím Nesúhlasím
1	EN	I do not worry much about visiting the dentist.	□	□
	CZ	Nemám moc velký strach z návštěvy zubaře.	□	□
	SK	Nezáleží mi na návštevách u zubného lekára.	□	□
2	EN	**My gum tends to bleed when I brush my teeth.**	□	□
	CZ	**Když si čistím zuby, moje dásně mají sklon krvácet.**	□	□
	SK	**Moje ďasná majú tendenciu krvácať pri čistení zubov.**	□	□
3	EN	I worry about the color of my teeth.	□	□
	CZ	Záleží mi na barvě mých zubů.	□	□
	SK	Záleží mi na farbe mojich zubov.	□	□
4	EN	**I have noticed some white sticky deposits on my teeth.**	□	□
	CZ	**Všiml(a) jsem si bílých lepivých nánosů na mých zubech.**	□	□
	SK	**Všimla/všimol som si biele usadeniny na mojich zuboch.**	□	□
5	EN	I use a child sized toothbrush.	□	□
	CZ	Používám zubní kartáček s malou hlavičkou.	□	□
	SK	Používam zubnú kefku detskej veľkosti.	□	□
6	EN	**I think that I cannot help having false teeth when I am old.**	□	□
	CZ	**Myslím si, že ve stáří budu nosit zubní protézy a nemůžu s tím nic dělat.**	□	□
	SK	**Myslím, že sa v budúcnosti nevyhnem noseniu protézy.**	□	□
7	EN	I am bothered by the color of my gum.	□	□
	CZ	Vadí mi barva mých dásní.	□	□
	SK	Trápi ma farba mojich ďasien.	□	□
8	EN	**I think my teeth are getting worse despite my daily brushing.**	□	□
	CZ	**Myslím si, že stav mých zubů se zhoršuje, i přesto, že si je každý den čistím.**	□	□
	SK	**Aj napriek dennému čisteniu zubov mám pocit, že sa stav mojich zubov zhoršuje.**	□	□
9	EN	**I brush each of my teeth carefully.**	□	□
	CZ	**Pečlivě si čistím každý zub zvlášť.**	□	□
	SK	**Čistím si poctivo každý zub.**	□	□
10	EN	**I have never been taught professionally how to brush.**	□	□
	CZ	**Nikdy jsem nebyl(a) odborně poučen(a), jak si mám čistit zuby.**	□	□
	SK	**Nikdy som neabsolvoval sedenie s hygienistkou ohľadom správnej techniky čistenia zubov.**	□	□
11	EN	**I think I can clean my teeth well without using toothpaste.**	□	□
	CZ	**Myslím si, že si mohu dobře vyčistit zuby i bez použití zubní pasty.**	□	□
	SK	**Myslím, že si viem dobre vyčistiť zuby bez použitia zubnej pasty.**	□	□
12	EN	**I often check my teeth in a mirror after brushing.**	□	□
	CZ	**Po čištění často kontroluji své zuby v zrcadle.**	□	□
	SK	**Často si kontrolujem zuby po vyčistení v zrkadle.**	□	□
13	EN	I worry about having bad breath.	□	□
	CZ	Mám obavy, že je mi cítit z úst.	□	□
	SK	Obávam sa halitózy.	□	□
14	EN	**It is impossible to prevent gum disease with tooth brushing alone.**	□	□
	CZ	**Není možné předcházet onemocnění dásní pouze pomocí čištění zubů.**	□	□
	SK	**Je nemožné predísť gingivitíde len s čistením zubov zubnou kefkou.**	□	□
15	EN	**I put off going to dentist until I have a toothache.**	□	□
	CZ	**Odkládám návštěvu zubního lékaře, dokud mě zuby nebolí.**	□	□
	SK	**Odkladám návštevu zubára až kým ma nezačnú bolieť zuby.**	□	□
16	EN	**I have used a dye to see how clean my teeth are.**	□	□
	CZ	**Použil(a) jsem barvící detektor plaku, abych si zkontroloval(a), jak jsou mé zuby vyčištěné.**	□	□
	SK	**Použil som v minulosti plak indikátor na zlepšenie orálnej hygieny.**	□	□
17	EN	I use a toothbrush which has hard bristles.	□	□
	CZ	Používám kartáček s tvrdými štětinami.	□	□
	SK	Používam zubnú kefku s tvrdými štetinami.	□	□
18	EN	I do not feel I have brushed well unless I brush with hard strokes.	□	□
	CZ	Nemám pocit vyčištěných zubů, pokud na kartáček hodně netlačím.	□	□
	SK	Nemám pocit čistých zubov pokiaľ netlačím na zubnú kefku.	□	□
19	EN	**I feel I sometimes take too much time to brush my teeth.**	□	□
	CZ	**Někdy mám pocit, že mi čištění zubů bere příliš mnoho času.**	□	□
	SK	**Mám pocit, že umývanie zubov mi zaberá príliš veľa času.**	□	□
20	EN	I have had my dentist tell me that I brush very well.	□	□
	CZ	Můj zubní lékař mi řekl, že si čistím zuby velmi dobře.	□	□
	SK	Zubný lekár ma pochválil za orálnu hygiene.	□	□
21	EN	I find myself using my smartphone/compute longer than I planned.	□	□
	CZ	Používám svůj smartphone nebo počítač déle, než jsem plánoval(a).	□	□
	SK	Používam svoj počítač alebo telefón dlhšie, než by som chcel.	□	□
22	EN	I consume tobacco at least once a week.	□	□
	CZ	Alespoň jednou týdně kouřím cigarety.	□	□
	SK	Minimálne jeden krát za týždeň užívam tabakové výrobky.	□	□
23	EN	I drink alcohol at least once a week.	□	□
	CZ	Alespoň jednou týdně mám alkoholický nápoj.	□	□
	SK	Minimálne jeden krát do týždňa pijem alkohol.	□	□
24	EN	I go to the dentist/ hygienist for regular check-up at least once a year.	□	□
	CZ	Alespoň jednou ročně navštěvuji zubního lékaře nebo dentální hygienistku.	□	□
	SK	Navštevujem zubného lekára/hygienistku minimálne jedenkrát za rok.	□	□

Items no. 1–20 are the original HU-DBI items, and the items in **bold** font are used to compute the overall score. The verb “worry” in item no. 1 was not translated equivalently in Czech and Slovak versions, and the term “child-sized toothbrush” in item no. 5 was not identical in both versions.
