# Supplementary material for: Czech and Slovak Dental Students’ Oral Health-Related Knowledge, Attitudes, and Behaviours (KAB): Multi-Country Cross-Sectional Study
Source: Int J Environ Res Public Health. 2022 Feb 25;19(5):2717. doi: 10.3390/ijerph19052717 (PMC8910048; doi:10.3390/ijerph19052717)
Supplement: Supplementary file 1 [file ijerph-19-02717-s001.zip › ijerph-1591770-supplementary.pdf]

Table S1. Test-re-test Reliability of HU-DBI Czech Version

Item	Response	First Round	Second Round	Cohen's κ
**No. 1**	Agree	32 / 40	33 / 40	0.754
**No. 2**	Disagree	34 / 40	33 / 40	0.908
**No. 3**	Agree	14 / 40	15 / 40	0.946
**No. 4**	Agree	9 / 40	10 / 40	0.931
**No. 5**	Agree	34 / 40	34 / 40	1.000
**No. 6**	Disagree	32 / 40	32 / 40	1.000
**No. 7**	Agree	3 / 40	3 / 40	1.000
**No. 8**	Disagree	29 / 40	30 / 40	0.935
**No. 9**	Agree	30 / 40	31 / 40	0.931
**No. 10**	Disagree	31 / 40	32 / 40	0.925
**No. 11**	Agree	21 / 40	22 / 40	0.950
**No. 12**	Agree	23 / 40	24 / 40	0.948
**No. 13**	Agree	15 / 40	14 / 40	0.946
**No. 14**	Disagree	25 / 40	27 / 40	0.781
**No. 15**	Disagree	30 / 40	30 / 40	1.000
**No. 16**	Agree	11 / 40	11 / 40	1.000
**No. 17**	Agree	4 / 40	5 / 40	0.875
**No. 18**	Agree	5 / 40	5 / 40	1.000
**No. 19**	Agree	16 / 40	16 / 40	1.000
**No. 20**	Agree	27 / 40	27 / 40	1.000
**Overall Cohen's κ**				0.941 ± 0.070
